# Supplementary material for: Expansion and Functional Diversification of SKP1-Like Genes in Wheat (Triticum aestivum L.)
Source: Int J Mol Sci. 2019 Jul 4;20(13):3295. doi: 10.3390/ijms20133295 (PMC6650978; doi:10.3390/ijms20133295)
Supplement: Supplementary file 1 [file ijms-20-03295-s001.zip › ijms-510341 final suppl/Figure S1.pdf]

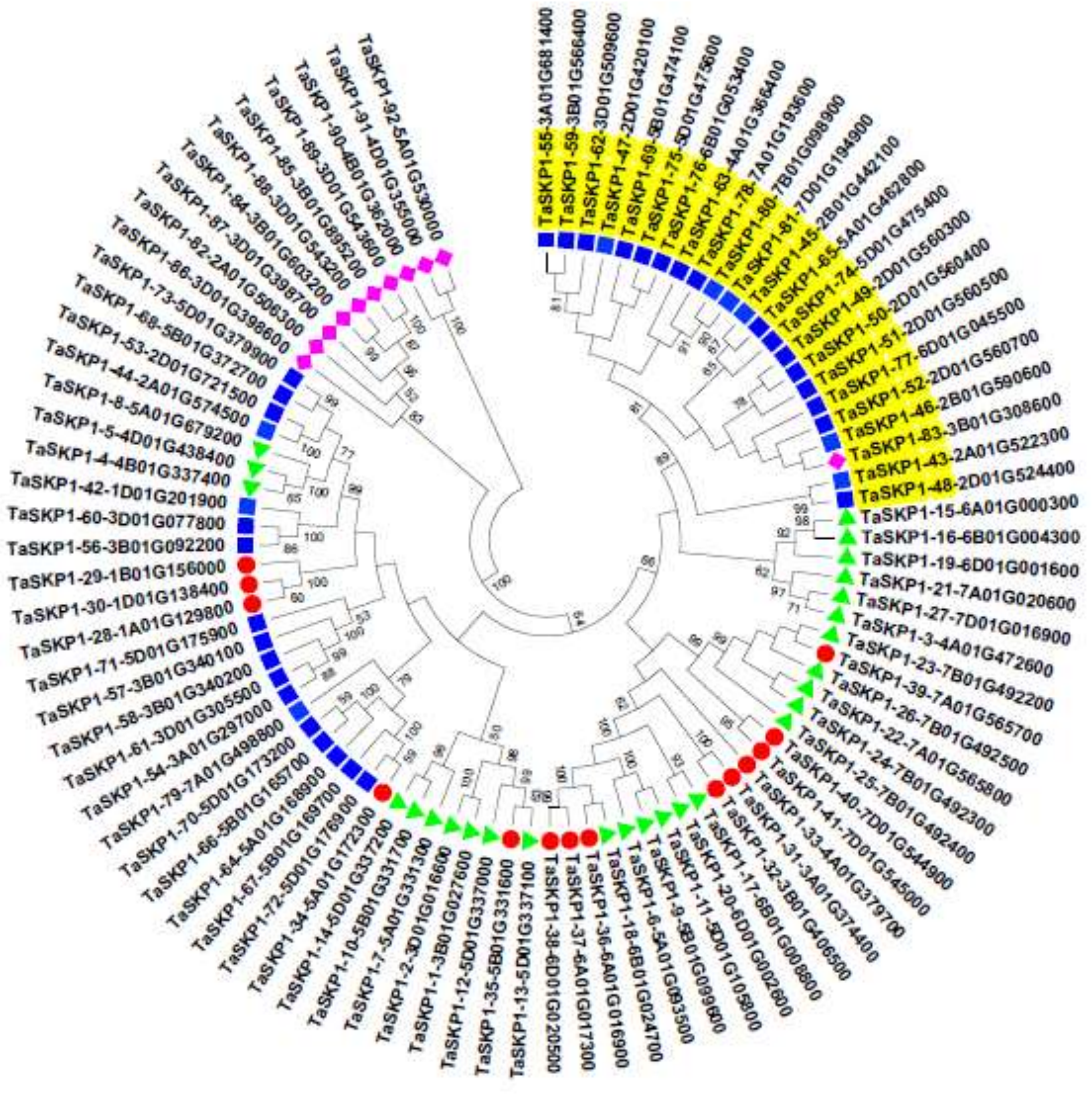

Figure S1. Blue squares represent *TaSKP1-like* intronless genes (Type Ib from Kong's groups, 1994). Red circles represent *TaSKP1-like* genes containing one non-conserved intron (Type Ia from Kong's groups, 1994). Pink diamonds represent *TaSKP1-like* genes containing more than one intron (Type II from Kong's groups, 1994). Green triangles represent *TaSKP1-like* genes containing one conserved intron (Type Ia from Kong's groups, 1994).
